# Rescue of a Vaccinia Virus Mutant Lacking IFN Resistance Genes K1L and C7L by the *Parapoxvirus* Orf Virus

**DOI:** 10.3389/fmicb.2020.01797

**Published:** 2020-07-28

**Authors:** Sherief Riad, Yan Xiang, Basheer AlDaif, Andrew A. Mercer, Stephen B. Fleming

**Affiliations:** ^1^Virus Research Unit, Department of Microbiology and Immunology, University of Otago, Dunedin, New Zealand; ^2^Department of Microbiology, Immunology and Molecular Genetics, University of Texas Health Science Center at San Antonio, San Antonio, TX, United States

**Keywords:** parapoxvirus, Orf virus, vaccinia virus, interferon, poxvirus, immune evasion

## Abstract

Type 1 interferons induce the upregulation of hundreds of interferon-stimulated genes (ISGs) that combat viral replication. The *parapoxvirus* orf virus (ORFV) induces acute pustular skin lesions in sheep and goats and can reinfect its host, however, little is known of its ability to resist IFN. Vaccinia virus (VACV) encodes a number of factors that modulate the IFN response including the host-range genes *C7L* and *K1L*. A recombinant VACV-Western Reserve (WR) strain in which the *K1L* and *C7L* genes have been deleted does not replicate in cells treated with IFN-β nor in HeLa cells in which the IFN response is constitutive and is inhibited at the level of intermediate gene expression. Furthermore C7L is conserved in almost all poxviruses. We provide evidence that shows that although ORFV is more sensitive to IFN-β compared with VACV, and lacks homologues of *KIL* and *C7L*, it nevertheless has the ability to rescue a VACV KIL- C7L- gfp+ mutant in which gfp is expressed from a late promoter. Co-infection of HeLa cells with the mutant and ORFV demonstrated that ORFV was able to overcome the block in translation of intermediate transcripts in the mutant virus, allowing it to progress to late gene expression and new viral particles. Our findings strongly suggest that ORFV encodes a factor(s) that, although different in structure to C7L or KIL, targets an anti-viral cellular mechanism that is a highly potent at killing poxviruses.

## Introduction

Type 1 IFNs are potent inhibitors of viral replication and are induced in most mammalian cells infected by viruses through Toll-like receptors, RIG-I/MDA5 RNA receptors and DNA sensors (Takaoka et al., 2007; Takeuchi and Akira, 2010; Jang et al., 2015). Type 1 IFNs induce an anti-viral state in neighboring cells via the janus kinase and signal activator of transcription (JAK/STAT) cell signaling pathway (Platanias, 2005; Sadler and Williams, 2008; Fensterl and Sen, 2009; Donnelly and Kotenko, 2010; MacMicking, 2012; Schneider et al., 2014). This leads to the upregulation of hundreds of interferon stimulated genes (ISGs) that inhibit viral replication (Schoggins and Rice, 2011; Schoggins et al., 2011; Schneider et al., 2014; Liu and Moss, 2018) at various stages of the virus life cycle including viral entry, replication, viral protein synthesis, and release (MacMicking, 2012; Schneider et al., 2014).

The first of the ISGs discovered were protein kinase activated by dsRNA (PKR) that inhibits protein synthesis by phosphorylation of eukaryotic translation initiation factor eIF2 (Zhang et al., 2001; Dar et al., 2005; Dey et al., 2005; García et al., 2007; Sadler and Williams, 2008; Dauber and Wolff, 2009; Johnston et al., 2012) and oligoadenylate synthetase (OAS), also activated by dsRNA, that specifically activate the latent form of endoribonuclease RNaseL leading to the degradation of viral RNA transcripts (Shors et al., 1997; Hovnanian et al., 1998; Hovanessian and Justesen, 2007; Sadler and Williams, 2008; MacMicking, 2012). There are several ISGs that block viral entry and uncoating. Interferon inducible transmembrane proteins (IFTM) IFITM1, IFITM2, and IFITM3 are constitutively expressed in many cells including epithelial cells at a basal level and upregulated by type I and type II IFNs (Mudhasani et al., 2013; Bailey et al., 2014; Li et al., 2018). IFITM proteins are enriched in late endosomes and lysosomes affecting viruses that require transit to these compartments for productive entry (Schoggins and Rice, 2011; MacMicking, 2012; Schneider et al., 2014).

Recently IFITM3 has been shown to inhibit vaccinia virus (VACV) infection by interfering with virus entry processes prior to the virus nucleocapsid entry into the cytoplasm (Li et al., 2018). MxA is a large, interferon-induced GTPase with antiviral activity (Rothman et al., 1990; Schwemmle et al., 1995; Janzen et al., 2000). MxA inhibits monkeypox virus but not VACV or cowpoxvirus (Lorenzo et al., 2017) and was shown to localize to the site of virus envelopment, although the mechanism of inhibition was not determined (Johnston et al., 2012). Sterile Alpha Motif Domain-containing 9 (SAMD9) and its close paralogue SAMD9L are two ISGs that were recently shown to play key roles in innate immune responses to viral infection (Lemos de Matos et al., 2013). SAMD9 was shown to inhibit late gene expression in cells infected with mutant myxoma virus (MYXV) (Liu and McFadden, 2015). A MYXV lacking the *M062R* gene was inhibited at the level of late gene expression by formation of SAMD9 granule structures in the cytoplasm near the viral factories (Liu et al., 2011; Liu and McFadden, 2015).

Members of *Poxviridae* family are large, complex double stranded DNA viruses that infect vertebrates and invertebrates (Traktman, 1996; Moss, 2007, 2013; Moss et al., 2007). Poxviruses are unusual in that they replicate and transcribe their large double stranded DNA genomes in the cytoplasm (Traktman, 1996; Moss, 2007, 2013; Moss et al., 2007). They have evolved sophisticated strategies to counteract the host response in particular the IFN response. VACV has been shown to encode multiple antagonists that allow it to inhibit the production of type 1 IFNs and modulate the JAK/STAT signaling pathway (Guan et al., 1991; Smith et al., 1998, 2013; Najarro et al., 2001; Mann et al., 2008; Koksal and Cingolani, 2011; Unterholzner et al., 2011; Smith, 2018), however, there have been few genes identified that encode factors that are involved in blocking the IFN effector response (Smith et al., 2018). K3L competes with activated PKR for interaction with e1F2α (Beattie et al., 1991; Davies et al., 1992; Carroll et al., 1993). *E3L* encodes a multifunctional factor that binds dsRNA and thus prevents the activation of PKR and inhibition of mRNA translation (Chang et al., 1992, 1995; Chang and Jacobs, 1993; Ho and Shuman, 1996; Langland and Jacobs, 2004). *C9L* encodes an ankyrin repeat /F-box protein that counteracts the action of IFN on early events of the replication cycle of VACV and interacts with the SCF (SKP, cullin1, F-box) complex and SCN (COP9 signalosome/deneddylation) complex (Liu and Moss, 2018). *C7L* and *KIL* have long been known as host range genes (Drillien et al., 1981; Gillard et al., 1986; Perkus et al., 1990) however, little was known about their role in pathogenesis until recently when it was revealed that KIL and C7L have roles in modulating the IFN effector response (Meng et al., 2009; Meng and Xiang, 2012). Deletion of both *K1L* and *C7L* genes render VACV sensitive to IFNs, which block the replication of the mutant virus at the step of translation of viral intermediate genes (Meng et al., 2009; Meng and Xiang, 2012). In particular the VACV mutant lacking *K1L* and *C7L* (vK1L^–^C7L^–^) was sensitive to the effects of interferon stimulated genes (Meng et al., 2009). vK1L^–^C7L^–^ replication in HeLa cells was also restricted (Perkus et al., 1990; Meng et al., 2012) correlating with their host range function. Furthermore SAMD9 was shown to be antagonized by K1L and C7L (Liu et al., 2011; Liu and McFadden, 2015; Sivan et al., 2015; Liem and Liu, 2016; Nounamo et al., 2017; Meng et al., 2018). SAMD9 expression inhibited the replication of a vK1L^–^C7L^–^ mutant and VACV viral factories were shown to colocalize with granules formed by SAMD9 (Liu and McFadden, 2015). Knocking down SAMD9 led to loss of the antiviral granules and allowed replication and late gene expression of vK1L^–^C7L^–^ (Liu and McFadden, 2015). Recently studies have shown that SAMD9 restricted the replication of poxvirus mutants that lack SAMD9 inhibitors through inhibition of viral protein synthesis, however, the mechanism of translational inhibition is unknown (Sivan et al., 2018). Meng et al., 2018 showed that SAMD9L functions similarly to SAMD9 as a restriction factor and that the two paralogues form a critical host barrier that poxviruses must overcome to establish infection. In HeLa cells SAMD9 and SAMD9L are poxvirus restriction factors (Meng et al., 2018). SAMD9 is constitutively active in HeLa cells while SAMD9L requires interferon induction (Meng et al., 2018). Although, knockout of SAMD9 was sufficient for abolishing the restriction for vK1L^–^C7L^–^ in many human cells, knockout of both paralogues was required for abolishing the restriction in interferon-treated cells (Liu and McFadden, 2015; Meng et al., 2018). K1L is only present in few *Orthopoxviruses* and expressed early during infection (Gillard et al., 1989; Assarsson et al., 2008; Meng et al., 2009; Yang et al., 2011). A functional homologue of C7L is present in almost all mammalian poxviruses (Oguiura et al., 1993; Assarsson et al., 2008; Meng et al., 2008, 2012; Yang et al., 2011).

Orf virus (ORFV) is a member of the *Parapoxvirus* genus (Buller and Palumbo, 1991; Moss, 2007; Hosamani et al., 2009; Fleming et al., 2015). It is a zoonotic virus that causes a highly contagious eruptive pustular skin disease in sheep and goats that can be transmitted to humans and other species (Haig and Mercer, 1998; Hosamani et al., 2009; Bergqvist et al., 2017). ORFV infects and replicates in keratinocytes cells, which are the major cell type of the epidermis (McKeever et al., 1988; Nestle et al., 2009; Fleming et al., 2015) and has not been reported to spread systemically. Genomic sequence and molecular analysis has revealed that ORFV encodes a number of factors that are likely to be involved in immune evasion such as anti-inflammatory factors including a viral homologue of cellular IL-10 and chemokine binding protein, and a factor that binds GM-CSF and IL-2 (Farner et al., 1997; Fleming et al., 1997, 2007, 2015; Deane et al., 2000; Wise et al., 2007). However, few investigations have been conducted to determine if ORFV can resist IFN. We recently showed that ORFV blocks the JAK/STAT IFN signaling pathway Harvey et al. (2015) and Haig et al. (1998) have shown that ORFV induces cytopathology in ovine cells pretreated with IFN-α and IFN-Ɣ. A homologue of VACV E3L encoded by ORFV binds dsRNA thus blocking the activation of PKR (Haig et al., 1998; Langland et al., 2006; García et al., 2007). We and others (Diel et al., 2010) have found that ORFV replicates in HeLa cells in which a vK1L^–^C7L^–^ mutant shows abortive replication (Meng et al., 2012). Interestingly ORFV does not encode homologues of K1L or C7L. In order to gain further insight into the ability of ORFV to resist IFN, we investigated whether ORFV could rescue a VACV recombinant deficient in *C7L* and *KIL* and expressing GFP from the late P11 promoter (vK1L^–^C7L^–^/GFP^+^). We showed that ORFV can provide some rescue to the vK1L^–^C7L^–^/GFP^+^ allowing it to progress to late gene expression and produce new viral particles.

## Materials and Methods

### Cells and Viruses

Primary Lamb Testis (LT) cells were grown at 37°C in minimal essential medium eagle (MEM) (Sigma) supplemented with 10% fetal calf serum (FCS) and 1% PSK antibiotic mix containing penicillin (500 units/ml), streptomycin (0.5 mg/ml) and kanamycin (0.1 mg/ml) (Robinson et al., 1982) Vero and HeLa cells were grown at 37°C in Dulbecco’s Modified Eagle Medium (DMEM) (Sigma) supplemented with 10% fetal calf serum (FCS) and 1% PSK antibiotic mix. 143B cells that are thymidine kinase deficient (TK-), ATCC^®^ CRL-8303^TM^ and resistant to BUdR were grown at 37°C in MEM supplemented with 10% FCS, 1% PSK antibiotic mix and 1% L-Glutamine. Primary Human Dermal Fibroblasts (HDF) were grown at 37°C in Dulbecco’s Modified Eagle Medium (DMEM) (Sigma) supplemented with 10 % FCS that was not heat inactivated and 1% PSK antibiotic mix. Recombinant VACV (Western Reverse) strains: recombinant virus in which the K1L and C7L genes are deleted and expresses GFP from a VACV late (P11) promoter (vK1L^–^C7L^–^/GFP^+^) (Meng et al., 2009) recombinant virus in which the K1L gene is deleted and the C7L gene is present and expresses GFP from a VACV late (P11) promoter (vK1L^–^C7L^+^/GFP^+^) (Meng et al., 2009, 2012). These recombinants were propagated in Vero Cells. VACV (Lister) and ORFV strain NZ2 (Robinson et al., 1982) were provided by the Virus Research Unit at the Department of Microbiology and Immunology, University of Otago.

### Virus Titration and Plaque Assay

The method for viral titration was adapted from Balassu and Robinson (1987). Virus titrations were carried out in a 6 well plate. Cells (LT for ORFV or TK-143B cells and VERO for VACV) were grown at 37°C in media containing 10% FBS and 1% PSK until they were 90% confluent. A 10-fold serial dilution of the viral preparation was made in PBS. The medium was removed, and the cells were washed with 1 mL of PBS. Two hundred microliters of the desired viral dilution were added to each well in duplicate. The infected cells were incubated at 37°C for 1 h for absorption with tipping every 10–15 min. After absorption, the inoculum was removed, and the cells were washed with 1 mL of PBS. Two milliliters of a 2% low melting point agarose overlay mixed 1:1 with 2× medium containing 4% FCS was added to each well. The agarose was left to set at RT and the infected cells were incubated at 37°C for 2 days for VACV or 5 days for ORFV. The plaques were visualized by staining with Neutral Red. A 2% low melting point agarose overlay was mixed with Neutral Red to give a final concentration of 0.015%.

### IFN Sensitivity Assays

HDFs were grown to 98% confluence in a 6-well culture plate at 37°C and treated with 0, 6.38, 63.8, 638, and 6380 U/mL of human IFN-β (PBL Assay Science) for 6 h and then infected with VACV-Lister or ORFV-NZ2 at an MOI of 0.01. The concentration of IFN used was based on findings described in Meng et al. (2009) and the units of activity of hIFN-β provided by the manufacturer. The low MOI was to allow multiple cycles of infection and determine the effect of IFN on the ability of the virus to go through multiple cycles of infection in the presence of an IFN effector response. The viruses were left 1 h for absorption at 37°C. The cells were washed with PBS and fresh medium containing 10% FCS was added. Cells infected with VACV-Lister were harvested at 24 hpi and cells infected with ORFV were harvested 72 hpi. Viral titres were determined using TK^–^143B cells for VACV-Lister and LT cells for ORFV.

### RT-PCR

HDFs were treated with hIFN-β as described above. The cells were then lysed, and total RNA was extracted using Total RNA Mini Kit (Geneaid). Reverse transcription-PCR (RT-PCR) was performed with SuperScript^TM^ IV VILO^TM^ Master Mix (Invitrogen^TM^). The following primers were used:

MX1 forward primer 5-TTCAGCACCTGATGGCCTATC-3 and reverse primer 5-TGGATGATCAAAGGGATGTGG-3, human glyceraldehyde-3-phosphate dehydrogenase (GAPDH) forward primer 5-CTCTGCTGATGCCCCCATGTTC-3 and reverse primer 5-GGTGGTGCAGGAGGCATTGCTG-3. MX1 and GAPDH primer sequences are described in (Harvey et al., 2015).

### Western Blot Analysis

Cell lysates (20 μl) were resolved by 12% SDS-polyacrylamide gel electrophoresis (SDS-PAGE) (Sambrook, 2001; Sambrook and Russell, 2006), transferred to nitrocellulose membranes and blocked with Tris-buffered saline and 0.1% Tween-20 (TBS-T) supplemented with 5% non-fat dried milk for 1 h at room temperature. Subsequently, membranes were incubated with the monoclonal or polyclonal antibody against the following proteins SAMD9 (Sigma), SAMD9L (Abcam), tubulin (abcam), MxA (Abcam), PKR (SANTA CRUZ^®^), IFIM3 (Abcam), tubulin (Abcam) overnight at 4°C. Next day, the membranes were washed with TBS-T and incubated with horseradish peroxidase-conjugated secondary antibodies (Amersham) and analyzed with chemiluminescence reagents according to the manufacturer’s instructions.

### vK1L^–^C7L^–^/GFP^+^ Rescue Assays and Flow Cytometry

HeLa cells were seeded in a 24 well plate at 2 × 10^5^ cells/well. Cells were grown at 37°C in media containing 10% FBS and 1% PSK until they were 90% confluent. The next day cells were infected with ORFV and vK1L^–^C7L^–^/GFP^+^. After absorption, the inoculum was removed, and cells were washed with 1 mL of PBS. Two milliliters of medium with 10% FCS was added to each well and cells were incubated at 37°C for 24 h. The next day, the medium was removed, the cells were washed with PBS and 1 mL of FACS buffer was added to all wells, after which cells were scraped off and the percentage of GFP positive cells was determined using BD FACSCanto. The data were analyzed by FlowJo^®^. A negative control for GFP expression in which cells were infected with ORFV only VACV-Lister only or cells only was used to set the gate for GFP positive cells and exclude background fluorescence

### Statistical Methods

All statistical analyses were performed using student’s *t*-test function from GraphPad prism version 6.00 for Mac, GraphPad Software, La Jolla California United States, www.graphpad.com. *p*-values less than 0.05 were considered significant.

## Results

### ORFV Is Relatively Sensitive to IFN-β Compared With VACV

**FIGURE 1:**
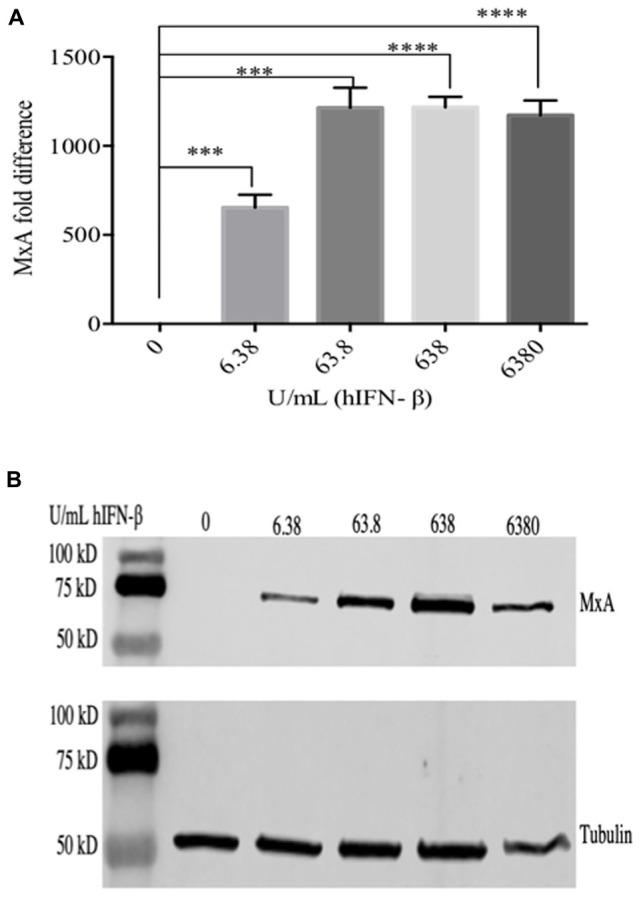
Cells were treated with hIFN-β at the concentrations shown for 6 h at 37°C. RNA was extracted and analyzed by RT-qPCR for MxA mRNA **(A)** and MxA protein was detected in cell lysates by western blot using anti-MxA antibody **(B)**. Anti-tubulin was used as a loading control. Error bars represent ± S.E.M from four independent experiments (^∗∗∗^*p* < 0.001, ^****^p < 0.0001, Student *T*-Test).

**FIGURE 2:**
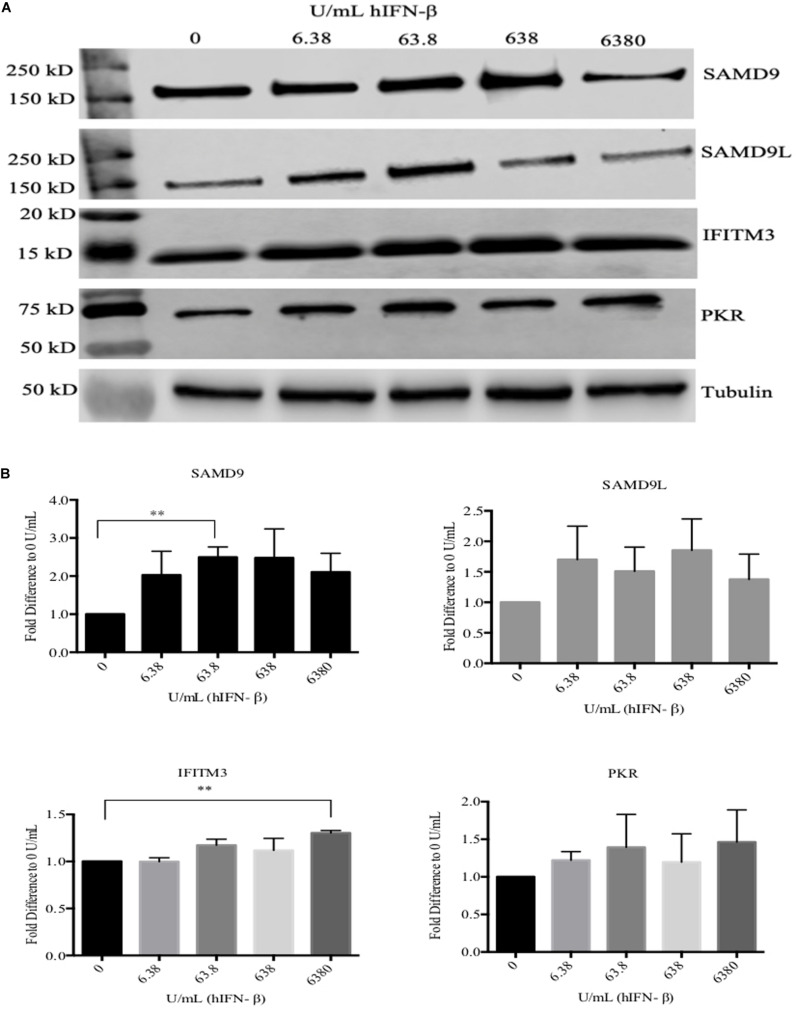
Western blot analysis of selected ISGs in HDFs treated with hIFN-β. Cells were treated with hIFN-β at the concentrations shown for 6 h. SAMD9, SAMD9L, IFITM3, and PKR were detected with their respective antibodies by Western blotting. Anti-tubulin antibody was used as a loading control **(A)**. The band intensity was quantified using Image Studio^TM^ then normalized to tubulin and further normalized to 0 U/mL of IFN **(B)**. Error bars represent ± SEM from three independent experiments (**p < 0.01, Student *T*-Test).

ORFV replicates in a number of primary cells lines that produce and are responsive to type I IFNs including HDFs (Supplementary Figure S1). We compared the ability of ORFV to replicate in IFN-β treated HDFs with VACV. Firstly, HDFs were treated with hIFN-β and the levels of selected ISGs investigated. *MxA* showed a 650-fold increase at the transcriptional level with 6.38 U/ml of IFN-β and more than a 1200-fold increase with higher amounts (63.8–6380 U/mL) (Figure 1). These results were consistent with protein analysis. MxA could not be detected in untreated cells but was detectable when cells were treated with 6.38 U/mL and increased with higher amounts of IFN (Figure 1). The polypeptide levels of SAMD9 showed a significant increase in response to IFN-β at 63.8 U/mL above untreated HDFs but only showed a further small increase with higher amounts of IFN-β (Figures 2A,B). SAMD9L showed a consistent increase to various amounts of IFN-β, however, this increase was not significantly greater than for untreated cells (Figures 2A,B). Basal levels of both IFITM3 and PKR were also detectable in untreated cells (Figure 2A). IFITM3 showed a small but significant increase in response to 6380 U/mL of IFN. PKR also showed an increase in response to IFN-β but was not significantly different to untreated cells (Figures 2A,B).

**FIGURE 3:**
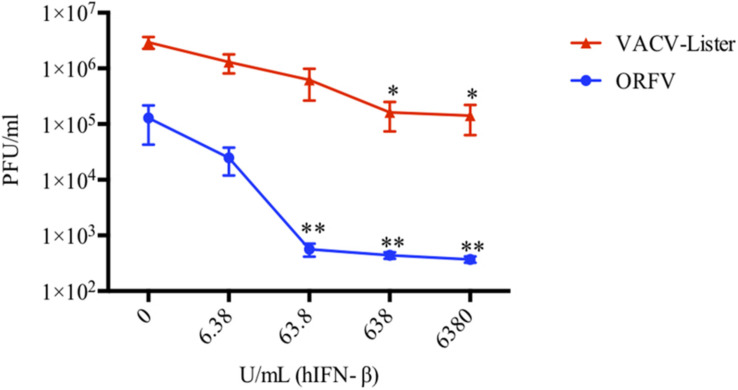
ORFV is more sensitive to hIFN-β than VACV. HDF cells were pre-treated with hIFN-β at the indicated concentrations for 6 h. Cells were then infected with either ORFV or VACV at an MOI of 0.01 and harvested at 24 hpi for VACV-Lister and 72 hpi for ORFV. The viral yields were determined by plaque assay on 143B cells for VACV and LT cells for ORFV. SEM and *p*-values were determined from four independent experiments where asterisks indicate a significant difference compared with no IFN treatment (*p < 0.05, **p < 0.01, Student *T*-Test).

Next, we investigated the ability of ORFV to replicate in HDFs treated with IFN-β and compared with *wt* VACV. We found that ORFV replication was only slightly affected by treatment with IFN at 6 U/mL, however, when cells were treated with higher amounts of IFN (>60 U/mL), replication was inhibited (Figure 3). In comparison, VACV was resistant to higher levels of IFN (60 U/mL) but showed reduced replication at levels above 600 U/mL. ORFV levels decreased by 5.2, 229, 293, and 348-fold at 6.38, 63.8, 638, and 6380 U/mL IFN-β, respectively. VACV decreased by 2.3, 4.7, 18, and 21-fold at 6.38, 63.8, 638, and 6380 U/mL IFN-β, respectively. These results clearly showed that ORFV is more sensitive to IFN than VACV.

### ORFV Replicates in HeLa Cells Despite Lacking Homologues of C7L and KIL

**FIGURE 4:**
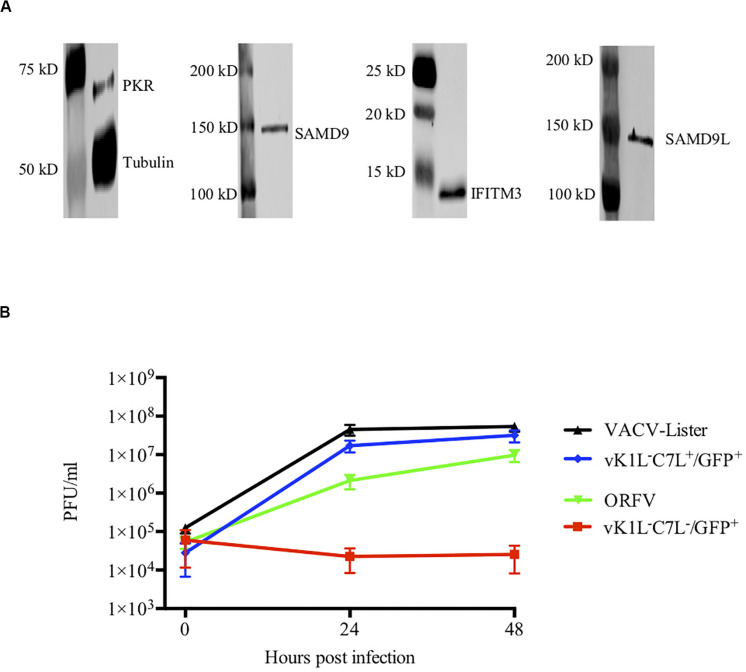
ORFV shows similar growth to VACV in HeLa cells. **(A)** Western blot analysis of interferon stimulated genes in HeLa cells. PKR, IFITM3, SAMD9, and SAMD9L were detected with their respective antibodies by Western blotting. Anti-tubulin antibody was used as a control. **(B)** HeLa cells were infected with either VACV-Lister, vK1L^–^C7L^+^/GFP^+^, vK1L^–^C7L^–^/GFP^+^, or ORFV at an MOI of 0.5. Viral titres were determined by serial dilutions using 143B cells for the VACV-Lister and LT cells for ORFV and Vero cells for vK1L^–^C7L^+^/GFP^+^ and vK1L^–^C7L^–^/GFP^+^. Virus plaques were visualized by staining with Neutral Red and counted.

VACV replicates in many mammalian cell lines including HeLa cells for which its host range genes C7L or KIL are essential. We and others have shown that HeLa cells are also permissive for ORFV. Although ORFV does not encode homologues of C7L or KIL our data suggests that ORFV may encode factors that act similarly to C7L or KIL. In order to verify the constitutive levels of ISGs in HeLa cells, we examined the levels of PKR, IFITM3, SAMD9 and SAMD9L during cell culture. We indeed showed that these molecules were expressed constitutively without IFN stimulation (Figure 4A). Note MxA is not expressed constitutively in HeLa cells and was not included in this analysis (Carey et al., 2008).

To gain further insight into its ability to replicate in HeLa cells we compared the growth of ORFV with *wt* VACV and a mutant VACV strain deficient in the C7L and KIL genes and expressing GFP from the late (P11) promoter vK1L^–^C7L^–^/GFP^+^. We found that ORFV replicated in HeLa cells almost as efficiently as VACV and as expected there was no evidence of replication of vK1L^–^C7L^–^/GFP^+^ in these cells (Figure 4B).

### ORFV Has the Ability to Rescue vK1L^–^C7L^–^/GFP^+^ Through to Late Gene Expression

**FIGURE 5:**
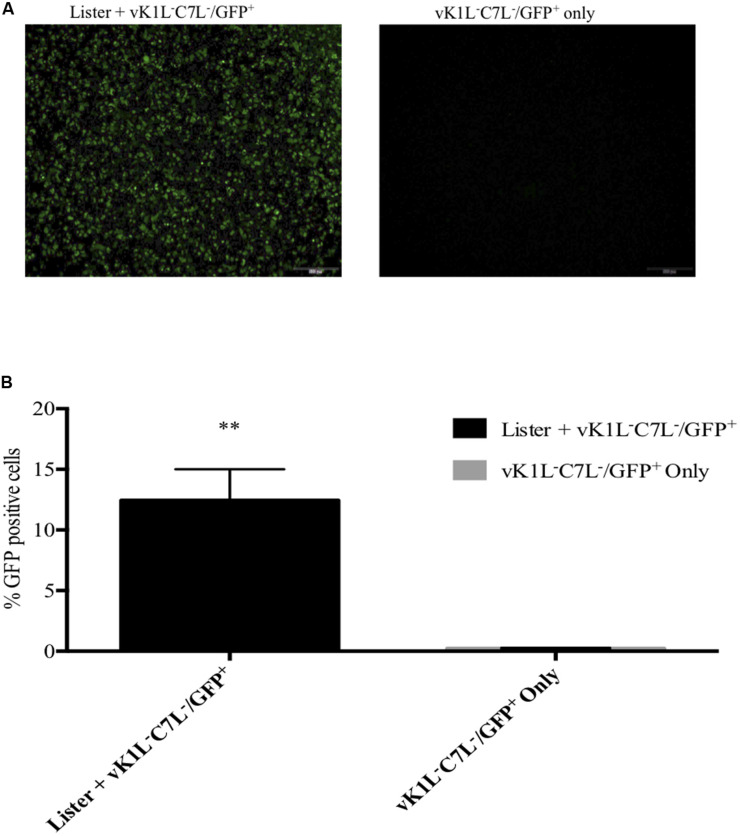
Partial rescue of vK1L^–^C7L^–^/GFP^+^ by VACV-Lister in HeLa cells. HeLa cells were co-infected with vK1L^–^C7L^–^/GFP^+^ and VACV-Lister each at an MOI of 1 and vK1L^–^C7L^–^/GFP^+^ only (MOI 1). At 8 hpi fluorescent microscopy images were taken at 4× magnification **(A)** and the percentage of GFP positive cells determined by FACS **(B)**. For each FACS sample, 10,000 events within the gated area were analyzed. The percentage of GFP positive cells for each group was compared to cells infected with vK1L^–^C7L^–^/GFP^+^ only. Error bars represent ± SEM from two independent experiments (**p < 0.01; Student’s *t*-test).

Our results suggested that ORFV might produce factors that have equivalent activity to VACV C7L and K1L although ORFV does not encode homologues of these genes. To test this hypothesis, we investigated the ability of ORFV to rescue vK1L^–^C7L^–^/GFP^+^. As a proof of concept, we firstly investigated if VACV rescued vK1L^–^C7L^–^/GFP^+^. The replication of the VACV mutant lacking K1L and C7L is inhibited at the step of intermediate viral gene mRNA translation in non-permissive cell lines (Hsiao et al., 2004; Meng et al., 2008, 2009). Since vK1L^–^C7L^–^/GFP^+^ expresses GFP from a VACV late promoter (P11), we hypothesized that *wt* VACV will be able to complement for the function of K1L and C7L and would allow the vK1L^–^C7L^–^/GFP^+^ to progress through its life cycle beyond intermediate gene translation leading to elevated levels of GFP expression. HeLa cells were co-infected with vK1L^–^C7L^–^/GFP^+^ and VACV-Lister each at an MOI of 1. At 8 h post infection (hpi) cells were analyzed by microscopy for GFP fluorescence and FACS analysis for the percentage of GFP positive cells. Evidence of partial rescue is clearly seen in the fluorescence images whereas there was no fluorescence seen in cells infected with vK1L^–^C7L^–^/GFP^+^ only (Figure 5A). FACS analysis showed that there were 13% GFP positive cells when cells were co-infected with vK1L^–^C7L^–^/GFP^+^ and VACV-Lister whereas there were very few GFP positive cells infected with vK1L^–^C7L^–^/GFP^+^ only (Figure 5B).

**FIGURE 6:**
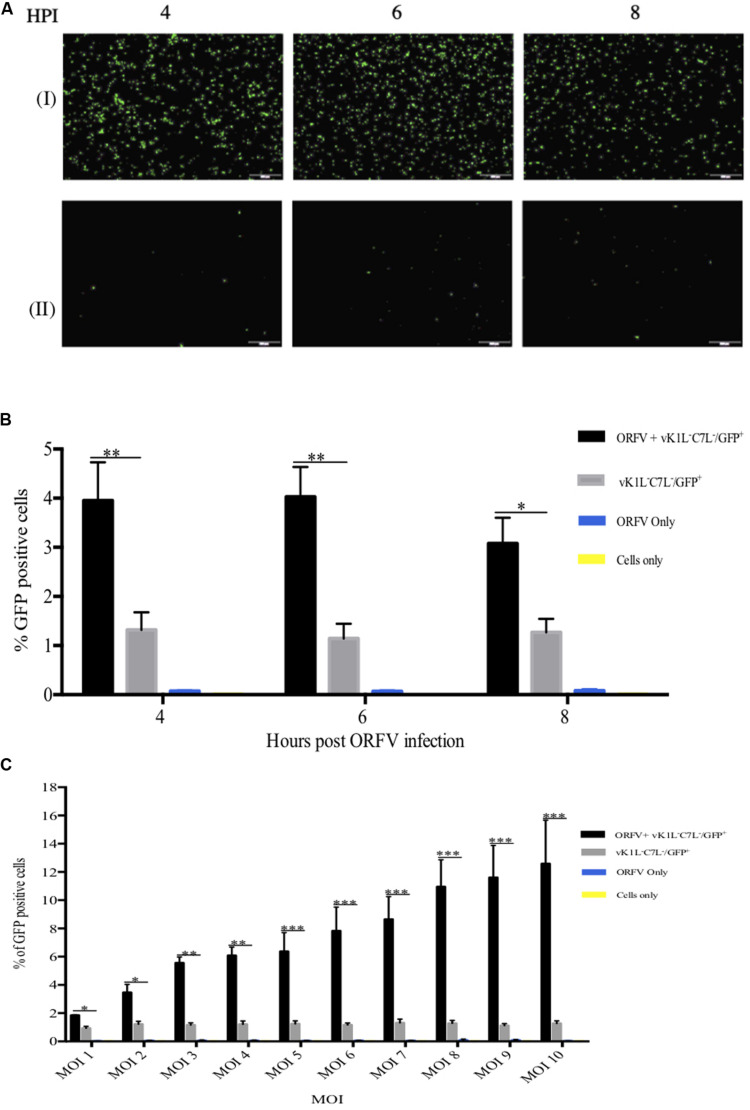
Partial rescue of vK1L^–^C7L^–^/GFP^+^ with ORFV. HeLa cells were co-infected with ORFV and vK1L^–^C7L^–^/GFP^+^ both at an MOI of 1 or infected with vK1L^–^C7L^–^/GFP^+^ only at an MOI of 1. Cells were firstly infected with ORFV and incubated for the times shown and then infected with the VACV mutant virus. At 24 h post VACV mutant infection GFP fluorescence images were taken at 4X magnification **(A)**. Cells co-infected with ORFV and vK1L^–^C7L^–^/GFP^+^ (I) or cells infected with vK1L^–^C7L^–^/GFP^+^ only (II). The percentage of GFP positive cells was determined by FACS **(B)**. For each FACS sample, 10,000 events within the gated area were analyzed. The percentage of GFP positive cells for each group was compared to cells infected with vK1L^–^C7L^–^/GFP^+^ only. A negative control for GFP expression in which cells were infected with ORFV only or cells only was used to set the gate for GFP positive cells and to exclude background fluorescence. Error bars represent ± S.E.M from four independent experiments (*p < 0.05, **p < 0.01; Student’s *t*-test). **(C)** HeLa cells were infected with ORFV for 6 h with an MOI of 1–10 and then infected with vK1L^–^C7L^–^/GFP^+^ also at an MOI of 1–10 i.e., the amount of ORFV and vK1L^–^C7L^–^/GFP^+^ were of equal MOIs at each titration. Cells were analyzed by flow cytometry at 24 h post vK1L^–^C7L^–^/GFP^+^ infection. Error bars represent ± SEM from four independent experiments (*p < 0.05,**p < 0.01, ***p < 0.001; Student’s *t*-test). A negative control for GFP expression in which cells were infected with ORFV only or cells only was used to set the gate for GFP positive cells and exclude background fluorescence.

We hypothesized that ORFV IFN resistance gene(s) will be expressed early, similar to C7L and K1L, to allow ORFV to overcome the IFN response. Therefore, HeLa cells were infected firstly with ORFV for 4, 6, and 8 h prior to infection with vK1L^–^C7L^–^/GFP^+^. Partial rescue of the vK1L^–^C7L^–^/GFP^+^ mutant by ORFV is clearly evident in the GFP fluorescence images at all time points (Figure 6A). The percentage of GFP positive cells was analyzed by flow cytometry (Figure 6B). The results show that 4–6 h was sufficient to provide partial rescue for the vK1L^–^C7L^–^/GFP^+^ mutant.

In order to optimize the rescue experiment titration experiments were performed. Figure 6C shows that with increasing amounts of ORFV and vK1L^–^C7L^–^/GFP^+^ higher rescue was achieved. At the highest MOI of ORFV (10) and the highest MOI of vK1L^–^C7L^–^/GFP^+^ (10) the percent positive GFP cells was 13% versus approximately 1% in cells infected with vK1L^–^C7L^–^/GFP^+^ only. Cells infected with ORFV only produced no fluorescence.

### ORFV Has the Ability to Rescue vK1L^–^C7L^–^/GFP^+^ in HeLa Cells

**FIGURE 7:**
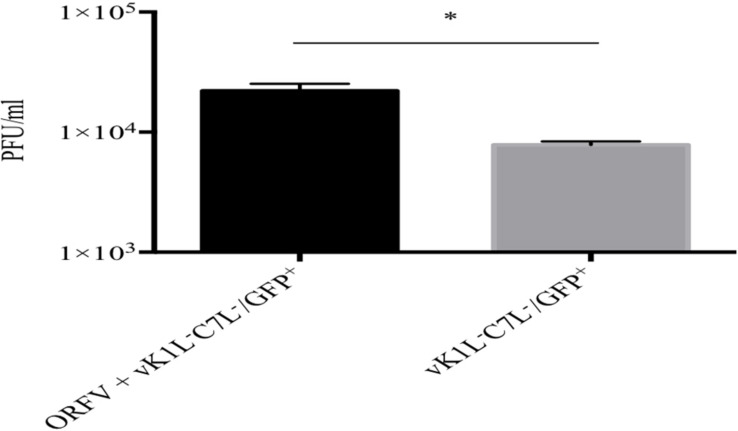
Rescue of vK1L**^–^**C7L**^–^**/GFP^+^ in HeLa cells by ORFV. HeLa cells were infected with ORFV at an MOI four and incubated for 6 h. The cells were washed and infected with vK1L**^–^**C7L**^–^**/GFP^+^ at an MOI of 1 and incubated for 24 h and then harvested. Viral titers were determined by serial dilution and infection of Vero cells. Virus plaques were visualized by GFP expression and staining with Neutral Red and counted. All plaques counted expressed GFP, Error bars represent ± SEM from three independent experiments (*p < 0.05 Student *T*-Test).

Our results thus far showed that ORFV has the ability to partially rescue a vK1L^–^C7L^–^/GFP^+^ mutant in HeLa cells allowing it to progress through its life cycle to late gene expression. We wished to determine if ORFV could provide full rescue to vK1L^–^C7L^–^/GFP^+^ and enable it to produce infectious viral particles. HeLa cells were co-infected with ORFV and vK1L^–^C7L^–^/GFP^+^ using an MOI of 4 and 1 for ORFV and vK1L^–^C7L^–^/GFP^+^, respectively. A plaque assay was performed using Vero cells to determine the viral output of vK1L^–^C7L^–^/GFP^+^. We observed a significant increase in cells co-infected with vK1L^–^C7L^–^/GFP^+^ and ORFV compared to cells infected with vK1L^–^C7L^–^/GFP^+^ only demonstrating the ability of ORFV to rescue the mutant (Figure 7). Importantly Vero cells did not support the growth of ORFV as shown in Supplementary Figure S2.

## Discussion

There is virtually nothing known about the ability of ORFV to resist the IFN response. The only gene that has been identified that appears to have a role in combating IFN is OV20L, an antagonist of PKR. The replication of ORFV in HeLa cells, in which a mutant of VACV lacking both *KIL* and *C7L*, suggested that ORFV may encode factors with equivalent functions for combating some anti-viral factors involved in the IFN effector response. This study compared the ability of ORFV with VACV to resist the IFN effector response and then investigated whether or not ORFV has the ability to rescue a VACV mutant lacking both the *KIL* and *C7L* genes.

Firstly, our investigation focused on assays performed with primary HDFs and the ability of ORFV to replicate in cells in which the IFN response was induced. Our results clearly showed that ORFV was relatively sensitive to IFN-β compared with VACV. At 63.8 U/mL of IFN-β, the replication of ORFV was completely blocked compared with VACV that could resist up to 6380 U/mL. These preliminary experiments also appeared to show a correlation between ISG levels in cells and the ability of ORFV to replicate. With increasing amounts of IFN-β we found a dose-response relationship in which MxA and SAMD9 peaked at 63.8 U/mL, the amount of IFN that blocked ORFV replication. In addition, an increase in PKR and SAMD9L was also apparent in the Western blots shown at this level of IFN stimulation. Notably VACV was completely resistant to cells induced with 63.8 U/mL of IFN. In this study we only examined a few ISGs likely to be relevant to poxvirus infection although further ISGs could be involved in combating these viruses. Nevertheless, our data clearly showed that VACV could combat the induction of an IFN effector response with as much as 6380 U/mL of IFN-β and clearly substantially more resistant to IFN than ORFV. Previous investigations by Haig et al. (1998) showed that ORFV is resistant to cells pretreated with IFN-α and IFN-γ, however, only cytopathology was measured and not virus output.

ORFV replicates in HeLa cells in which we, and others (Diel et al., 2010) have shown that the IFN effector response is constitutive. PKR, IFITM3, SAMD9, and SAMD9L were all detected in these cells at the protein level and our results clearly showed that ORFV replicated to similar levels as VACV. Notably a VACV mutant lacking both *KIL* and *C7L* did not replicate, which is in agreement with published observations (Perkus et al., 1990; Meng et al., 2009, 2012; Meng and Xiang, 2012). Our data suggests that ORFV may have the capability to combat the same ISGs as VACV C7L. Interestingly SAMD9 and SAMD9L, critical targets of both KIL and C7L, could be detected in HeLa cells and our data suggests that ORFV can combat at least constitutive levels of these ISGs whereas replication of the VACV KIL^–^C7L^–^ mutant is completely inhibited. If ORFV produces factors that combat the same anti-viral molecules as C7L and KIL we hypothesized that ORFV could potentially rescue the VACV KIL^–^C7L^–^ mutant.

When comparing the ISG levels in HDFs stimulated with IFN and constitutive levels in HeLa cells it appears in fact that SAMD9, SAMD9L, IFITM3, and PKR are expressed at similar levels. Curiously MxA is not expressed in HeLa cells and was not detected in HDFs until stimulated with IFN-β. Interestingly in HDFs stimulated with IFN-β, the peak levels of MxA correlates with complete ORFV growth inhibition. As noted above, MxA inhibits monkeypox and our data might suggest that it inhibits ORFV. Overexpression of MxA in ORFV infected HeLa cells may help toward answering this question.

The use of one virus to rescue another by co-infection has been established since the 1960s. Research has shown that heat inactivated poxviruses can be reactivated using other infectious members of the poxvirus family (Hanafusa et al., 1959; Fenner and Woodroofe, 1960). Therefore, we investigated whether ORFV could provide rescue to vK1L^–^C7L^–^/GFP^+^ in HeLa cells. Although ORFV and VACV vary in duration of their replication cycles and temporal expression of genes, we were able to demonstrate that ORFV was able to rescue the mutant through to late gene expression by infecting cells firstly with ORFV to allow expression of the early genes and then with VACV. Notably ORFV did not appear to encode any factors that led to the exclusion of VACV in ORFV infected cells. Although VACV could infect ORFV infected cells there was the possibility that ORFV infection compromised the ability of VACV to complete its life cycle. The data indicated that this could be the case as the intensity of GFP expression for co-infection by ORFV and vK1L^–^C7L^–^/GFP^+^ was less than for simultaneous co-infection with VACV *wt* and vK1L^–^C7L^–^/GFP^+^. Even so our results indicated that ORFV expressed factors that were able to overcome the block in VACV intermediate transcript translation. Our data also showed that ORFV progressed the life cycle of vK1L^–^C7L^–^/GFP^+^ through to the production of new infectious virus particles. Although the numbers of new viral particles were significantly more than for vK1L^–^C7L^–^/GFP^+^ alone, they were less than two-fold. These findings are consistent with our observations above that suggest that although ORFV produces factors that complement KIL and C7L, they are clearly less potent in their ability to neutralize the IFN effectors targeted by these factors.

Skin is the largest organ of vertebrates and is highly susceptible to infection by microbes through cuts and abrasions. A complex immunological system has evolved in skin (Nguyen and Soulika, 2019) to combat infection and elicits a potent IFN response. Yet, our results suggest that ORFV is relatively sensitive to the effects of IFN compared with other poxviruses. ORFV replicates exclusively in keratinocytes in its natural hosts and these cells are considered as immunological sentinels. Keratinocytes express TLRs, located either on the cell surface or in endosomes, in addition to TLR7 the expression of which is induced through triggering of TLR3 by dsRNA (Lebre et al., 2007; Nestle et al., 2009). Keratinocytes respond to pathogens by producing cytokines, chemokines and type I IFNs (Miller and Modlin, 2007; Nestle et al., 2009; Bangert et al., 2011) that include the proinflammatory cytokines IL-1, IL-6, IL-8, IL-18 and tumor necrosis factor (TNFα) (Grone, 2002; Tuzun et al., 2007; Bangert et al., 2011; Turner et al., 2014) and Torseth et al. and others (Schnipper et al., 1984; Torseth et al., 1987) have shown that IFN-β is produced by keratinocytes in response to Herpes simplex virus infection. Furthermore, plasmacytoid DC are found in the dermal layer of skin (Nestle et al., 2009; Swiecki and Colonna, 2011) and secrete large amounts of type I IFNs and it has been suggested that pDCs contribute to antiviral responses against viral infections of the skin (Gerlini et al., 2006; Swiecki and Colonna, 2011; Vermi et al., 2011). pDC infiltrate human skin lesions and produce type I IFNs during virus infections caused by Molluscum contagiosum virus (MCV) and Varicella zoster virus (Gerlini et al., 2006; Swiecki and Colonna, 2011; Vermi et al., 2011). ORFV has been shown to encode a range of molecules to combat inflammation and the immune response including a viral IL-10 (Fleming et al., 1997, 2007), chemokine binding protein (Seet et al., 2003), NFκB inhibitors (Diel et al., 2010, 2011a, b) and a factor that binds granulocyte macrophage colony stimulating factor and IL-2 (Deane et al., 2000). In addition, ORFV encodes a number of novel genes within the terminal regions of the genome for which functions have yet to be discovered and it’s likely that some may be involved in combating the host response (Fleming and Mercer, 2007; Fleming et al., 2015). ORFV can persist in the skin for 6–8 weeks post-infection that strongly suggests that ORFV has evolved strategies to combat the IFN response. We and others have shown that it has the ability to potently inhibit the JAK/STAT pathway and type I IFN production (unpublished data) and a factor OV20.0 that blocks the activity of PKR and this study suggests that there are further factors. Intriguingly ORFV does not encode homologues of secreted IFN receptor-like factors which are produced by many other poxviruses (Mercer et al., 2006; Fleming, 2016).

The absence of a clear homologue for *C7L* or *K1L* genes within the ORFV genome or a factor that is as potent as C7L or K1L possibly restricts ORFV’s ability to cause systemic infection. Notably, genetic analysis of the poxvirus MCV, that also replicates only in human skin and induces a low-grade persistent infection, has revealed that it too does not encode homologues of C7L or KIL (Meng and Xiang, 2006; Meng et al., 2012). Keratinocytes express SAMD9 (Gillbro et al., 2014) suggesting that ORFV and MCV have evolved strategies to combat its anti-viral activity. Interestingly the human papilloma virus oncoprotein E6 has been shown to interact with SAMD9 in an immortalized keratinocyte cell line (Wang et al., 2016). Our results suggest that ORFV has a mechanism to mitigate at least low levels of SAMD9 such as that found in HeLa cells and it’s possible that MCV has a similar mechanism. In addition, ORFV may also have strategy to diminish the expression levels of SAMD9 and its paralogue within the immune environment of localized highly inflamed skin pustules by subverting the effects of IFN. Such a mechanism could partly explain the tropism (restriction) of this virus to skin.

The evolution of poxvirus genomes has been remarkably dynamic. Although the gene arrangement has been strongly conserved, particularly the core essential genes, there has been substantial gene loss and gain throughout poxvirus evolution (McLysaght et al., 2003). Several researches have shown that poxvirus genomes have evolved by gene duplication, recombination between genes, horizontal gene transfer and gene loss and gain (McLysaght et al., 2003; Hughes and Friedman, 2005; Hendrickson et al., 2010). These changes played a critical role in speciation of viruses to a particular environmental (host or tissue) niche (Hendrickson et al., 2010). Since ORFV does not cause systemic infection (Haig and McInnes, 2002) it is possible that it lost its C7L homologue but maintained a gene that enables it to resist the IFN effector response but to a lesser degree than VACV and better suited for its growth in keratinocytes within skin tissue. Surprisingly, VACV was found to have limited replication in keratinocytes (Liu et al., 2005). The variability between different *Orthopoxvirus* genomes is most apparent in genes that are functionally involved in various virus-host interactions (Hendrickson et al., 2010). McLysaght et al. (2003) showed that *Orthopoxvirus* genomes have high rates of gene acquisition which is indicative of adaptive genome evolution as many of these acquired genes are associated with host–parasite co-evolution a feature that is also emerging in *Parapoxviruses*.

In conclusion, molecular studies of VACV and our findings from this study strongly suggest that ORFV has a strategy to block the IFN effector response that does not require the potency of VACV. ORFV has adapted to the skin and replication in keratinocytes by expressing a unique set of factors that does not include proteins structurally related to C7L or KIL (Fleming et al., 2015). Our findings strongly suggest that ORFV encodes a factor(s) that, although different in structure to C7L or KIL, target an anti-viral cellular mechanism that is a highly potent at killing poxviruses, indicative of convergent evolution. Future studies could involve examining the specificity of ORFV on SAMD9 activity, investigating the effects of SAMD9/SAMD9L knockdown or over expression on ORFV replication, screening ORFV proteins for their ability to rescue the VACV K1L C7L deletion mutant, gene screening to identify ORFV proteins that directly interact with SAMD9 using immunoprecipitation and mass spectroscopy.

## Data Availability Statement

The raw data supporting the conclusions of this article will be made available by the authors, without undue reservation.

## Author Contributions

SF conceived the study. SR and SF designed the experiments and wrote the manuscript. SR and BA performed the study. SF and AM supervised the research. YX provided the VACV recombinant viruses. All authors contributed to the article and approved the submitted version.

## Conflict of Interest

The authors declare that the research was conducted in the absence of any commercial or financial relationships that could be construed as a potential conflict of interest.
