# Supplementary figures and images for: Rescue of a Vaccinia Virus Mutant Lacking IFN Resistance Genes K1L and C7L by the Parapoxvirus Orf Virus
Source: Front Microbiol. 2020 Jul 28;11:1797. doi: 10.3389/fmicb.2020.01797 (PMC7438785; doi:10.3389/fmicb.2020.01797)

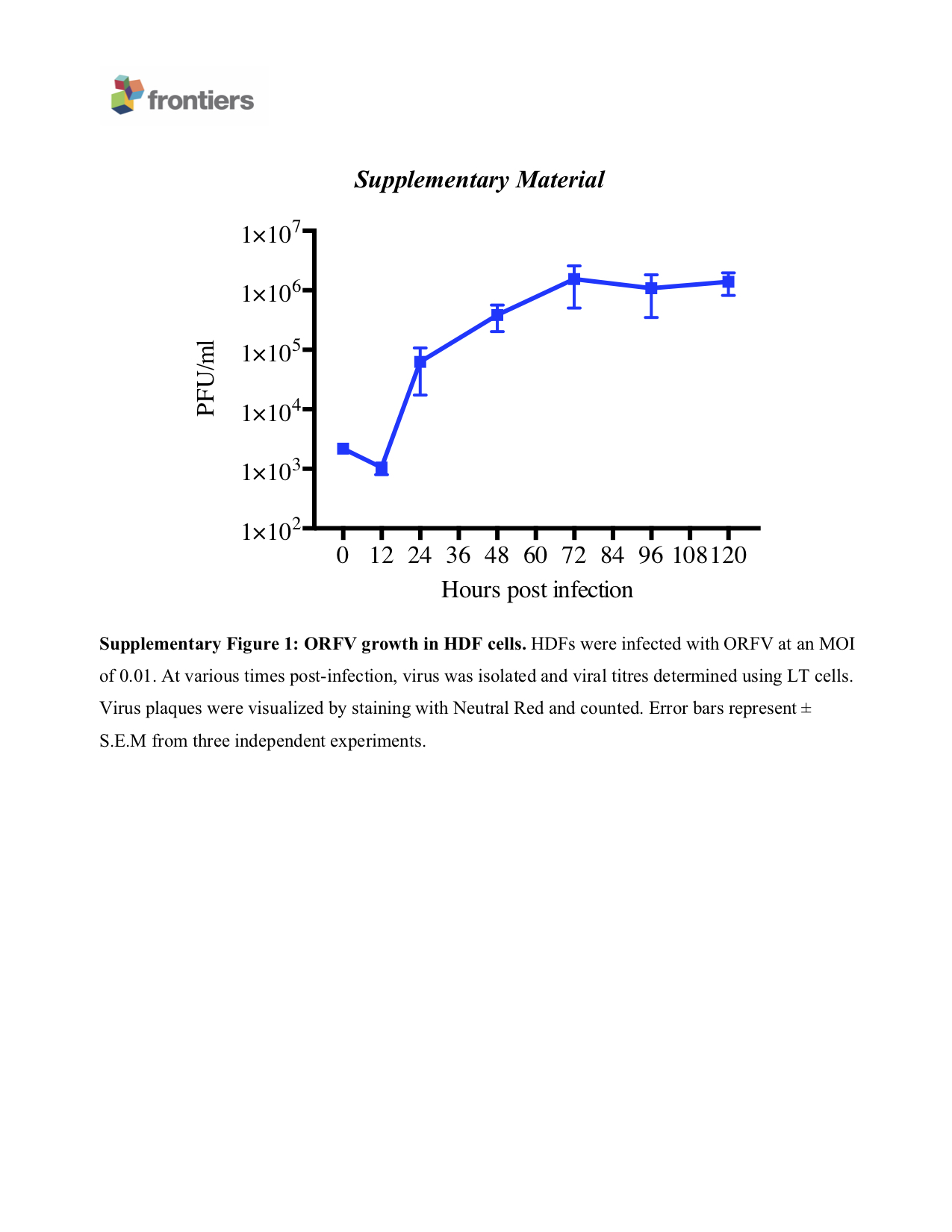

Supplement: Supplementary file 1 [file Image_1.JPEG]

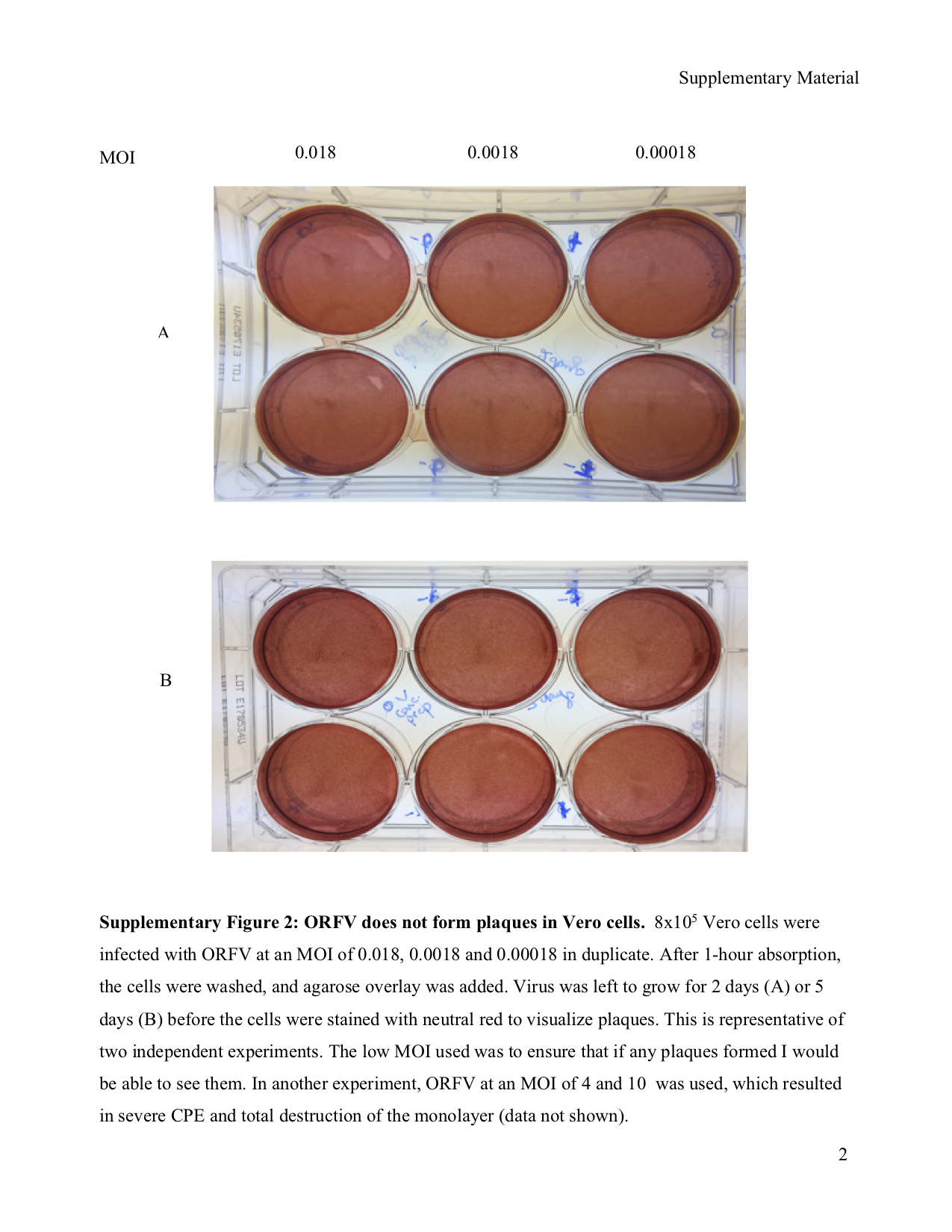

Supplement: Supplementary file 2 [file Image_2.JPEG]
